# *In vitro* assessment of pancreatic hormone secretion from isolated porcine islets

**DOI:** 10.3389/fendo.2022.935060

**Published:** 2022-08-11

**Authors:** Nizar I. Mourad, Daela Xhema, Pierre Gianello

**Affiliations:** Pôle de chirurgie expérimentale et transplantation, Université catholique de Louvain, Brussels, Belgium

**Keywords:** porcine islets, glucagon, insulin, perifusion, secretion

## Abstract

The potential use of porcine islets for transplantation in humans has triggered interest in understanding porcine islet physiology. However, the number of studies dedicated to this topic has remained limited, as most islet physiologists prefer to use the less time-consuming rodent model or the more clinically relevant human islet. An often-overlooked aspect of pig islet physiology is its alpha cell activity and regulation of its glucagon secretion. *In vitro* islet perifusion is a reliable method to study the dynamics of hormone secretion in response to different stimuli. We thus used this method to quantify and study glucagon secretion from pig islets. Pancreatic islets were isolated from 20 neonatal (14 to 21-day old) and 5 adult (>2 years) pigs and cultured in appropriate media. Islet perifusion experiments were performed 8 to 10 days post-isolation for neonatal islets and 1 to 2 days post-isolation for adult islets. Insulin and glucagon were quantified in perifusion effluent fractions as well as in islet extracts by RIA. Increasing glucose concentration from 1 mM to 15 mM markedly inhibited glucagon secretion independently of animal age. Interestingly, the effect of high glucose was more drastic on glucagon secretion compared to its effect on insulin secretion. *In vivo*, glucose injection during IVGTT initiated a quick (2-10 minutes) 3-fold decrease of plasmatic glucagon whereas the increase of plasmatic insulin took 20 minutes to become significant. These results suggest that regulation of glucagon secretion significantly contributes to glucose homeostasis in pigs and might compensate for the mild changes in insulin secretion in response to changes in glucose concentration.

## Introduction

Pancreatic islets adapt their hormone output to surrounding glucose concentration through stimulus-secretion coupling mechanisms that translate glucose and other nutrient metabolism into increasing or decreasing secretory rates of the two major pancreatic hormones, insulin and glucagon. Beta-cells have been thoroughly studied for years and pathways by which glucose controls insulin secretion are mostly well-understood: glucose accelerates beta-cell metabolism resulting in an increase of cytosolic ATP/ADP ratio leading to closure of ATP-dependent potassium channels, membrane depolarization and opening of voltage-gated calcium channels which then triggers insulin granule exocytosis that can be further modulated by yet unknown metabolic signals resulting in amplification of glucose-induced insulin secretion thought to account for as much as 50% of total insulin response (1, 2). The situation is less clear in the case of alpha-cells as two major hypothesis have been debated (3): in a beta-cell-like model, glucose metabolism causes closure of ATP-dependent potassium channels but fails to sufficiently depolarize the membrane to open L-type calcium channels (4, 5) while inactivating voltage-dependent Na^+^ channels and therefore reducing Ca^2+^ entry *via* P/Q calcium channels due to diminished amplitude of action potentials (6, 7). This glucose-induced fall in [Ca^2+^]_i_ then suppresses glucagon secretion (8). Another model proposes ER store-operated calcium entry and subsequent membrane depolarization followed by further calcium entry *via* voltage-gated calcium channels as the mechanism by which low glucose stimulates glucagon secretion (9, 10). All of the above-mentioned studies utilized mostly rodent and sometimes human islet cells to explore these stimulus-secretion coupling mechanisms. In porcine islets, glucose has been shown to trigger biphasic insulin secretion with a major difference in the amplitude of the response as we (11) and others (12, 13) have reported a modest 2-4-fold increase of insulin secretion (11) upon stimulation with 15 mM glucose compared to a 10-fold increase at least following similar stimulation of human islets (14). Even less is known of the regulation of glucagon secretion from porcine islets with one study reporting very low basal and arginine-stimulated glucagon secretion from adult pig islets (15) while other studies focusing on porcine islet function have simply overlooked this aspect of their physiology. In the present study, we used *in vitro* dynamic perifusion of isolated islets as well as *in vivo* glucose tolerance test to characterize glucagon secretion from neonatal and adult porcine islets since they currently represent one of the most realistically relevant options for islet replacement therapy in the clinic (16).

## Materials and methods

### Islet isolation

All experiments were conducted in accordance with the local ethical committee and with EU Directive 2010/63/EU for animal experiments. Outbred pigs (Belgian landrace; 200-300 kg) were provided by Rattlerow-Seghers genetics (Ooigem, Belgium). Piglets (Belgian landrace; 3-8 kg) were directly delivered to the islet isolation facility in Brussels. Adult pigs were housed in the A. de Marbaix center (Louvain-la-Neuve, Belgium). Pancreatic islet isolation was carried out as previously described (11). Briefly, collagenase V from Sigma (Darmstadt, Germany) was used for piglet pancreas digestion and collagenase NB8 from Serva (Heidelberg, Germany) was used for adult pancreas digestion. Neonatal pig islets (NPI) were cultured in HAM-F10 culture medium supplemented with 0.5% BSA, 50 µM IBMX (3-isobutyl-1-methylxanthine), 10 mM glucose, 2 mM glutamine, 10 mM nicotinamide, 1% penicillin and 1% streptomycin during a period of 8 days to allow maturation of islet cells and removal of exocrine tissue as previously described (17). Adult porcine islets (API) were purified using a discontinuous Ficoll gradient before culture in RPMI 1640 supplemented with 10% heat-inactivated FBS, 1% penicillin, 1% streptomycin, 5 mM glutamine and 5 mM glucose for 12-24 hours before islet perifusion.

### *In vitro* insulin and glucagon secretion assays

Secretory function of isolated neonatal and adult islets was studied by dynamic islet perifusion experiments. the working medium was a bicarbonate-buffered solution containing 120 mM NaCl, 4.8 mM KCl, 2.5 mM CaCl_2_, 1.2 mM MgCl_2_, 24 mM NaHCO_3_, 1 mg/mL BSA and varying concentrations of glucose and test substances as indicated in the figures. Batches of 200-1000 IEQ were placed in perifusion chambers, covered with 8 µm cellulose filters and sealed. Test solutions kept at 37°C and gassed continuously to stabilize pH around 7.2 were pumped at a flow rate of 1 mL/min. Effluent fractions were collected at 2-minute intervals and saved for insulin and glucagon assays using radioimmunoassay (RIA) kits (Millipore, Billerica, MA, USA). At the end of the experiments, islets were recovered and their insulin and glucagon content was determined after extraction in acid-ethanol (75% ethanol, 180 mM HCl from Merck).

### Intravenous glucose tolerance test

To study *in vivo* secretion of insulin and glucagon in adult pigs, we performed glucose tolerance tests by administering an intravenous glucose bolus (0.5 g/kg bodyweight) in an auricular vein. Blood samples were taken before and at 1, 3, 5, 10, 20, 30, 60 and 90 minutes after glucose injection using a catheter placed on the other ear. Blood glucose was immediately measured using Aviva nano glucometer (Accu-Chek) and serum samples were prepared and stored at -80°C for subsequent insulin and glucagon assays.

## Results

### Glucose inhibits glucagon and stimulates insulin secretion from porcine islets

**Figure 1:**
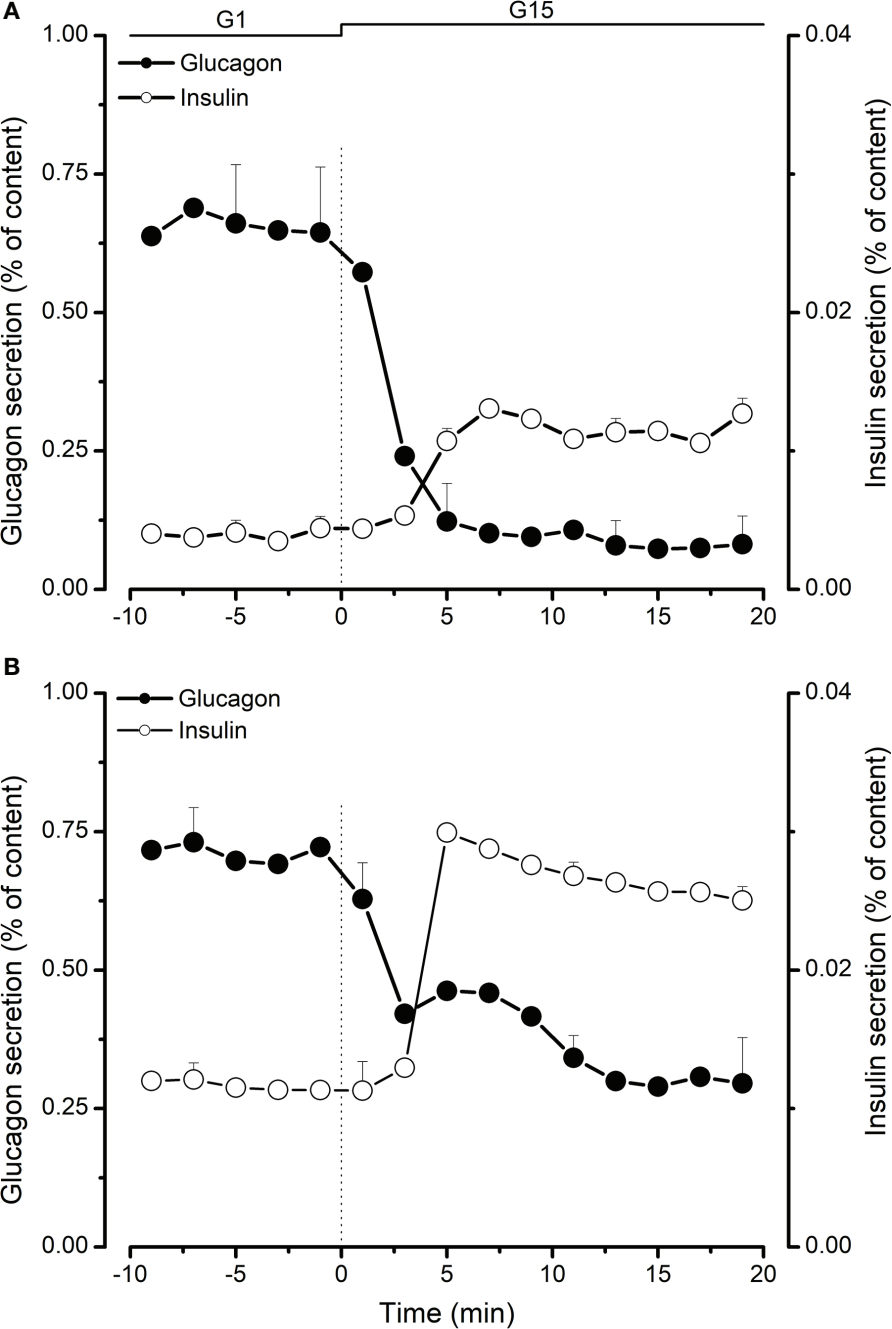
Comparison of glucagon (●) and insulin (○) secretion from neonatal **(A)** and adult **(B)** perifused porcine islets. Islets in 1 mM glucose (G1) were stimulated with 15 mM glucose (G15) for 20 minutes. Glucagon and insulin secretion was measured in effluent fractions and expressed as percentage of total glucagon or insulin islet content. Values are means ± SEM from n=4-8 different preparations from each age group.

Islet hormone secretion was tested after 8 days of culture in a maturation medium for neonatal islets and after 24-48 hours for adult islets. As shown in **Table 1**, islet glucagon content decreased from 817 pg/IEQ at neonatal age (2-3 weeks) to 471 pg/IEQ at adult age (>18 months). In parallel, insulin content increased from 113 µU/IEQ to 723 µU/IEQ at neonatal and adult age respectively. Using dynamic islet perifusion, we studied glucagon and insulin secretion in response to changes in glucose concentration. At low glucose (1 mM; G1), glucagon secretion was stimulated in both neonatal (2.6 pg/IEQ/min; **Figure 1A**) and adult (1.5 pg/IEQ/min; **Figure 1B**) islets. Basal insulin secretion was lower in neonatal islets compared to adult islets, 2.10^-3^ µU/IEQ/min vs. 4.10^-2^ µU/IEQ/min respectively. Upon stimulation with 15 mM glucose (G15), glucagon secretion dropped 6-fold in neonatal islets and only 2-fold in adult islets. In parallel, insulin secretion increased similarly in both neonatal and adult islets (2.6- and 2.3-fold respectively; **Figures 1A**, **B**). Interestingly, inhibition of glucagon secretion occurred after 4 minutes of glucose stimulation whereas the increase of insulin secretion took 6 minutes to become measurable as shown in **Figure 1**.

**Table 1:** Glucagon and insulin contents of neonatal and adult isolated porcine islets.

	Glucagon (pg/IEQ)	Insulin (µU/IEQ)
Neonatal	817 ± 89	113 ± 16
Adult	471 ± 55	723 ± 102

Islet hormone content was calculated from lysates containing 30 islets. Islets were hand picked then incubated in acid-ethanol at -20°C for 12 hours at least. Cell disruption was then completed by sonication. Values are means ± SD from 5 lysates for each age group.

### Role of ATP-dependent potassium channels in regulation of glucagon secretion

**Figure 2:**
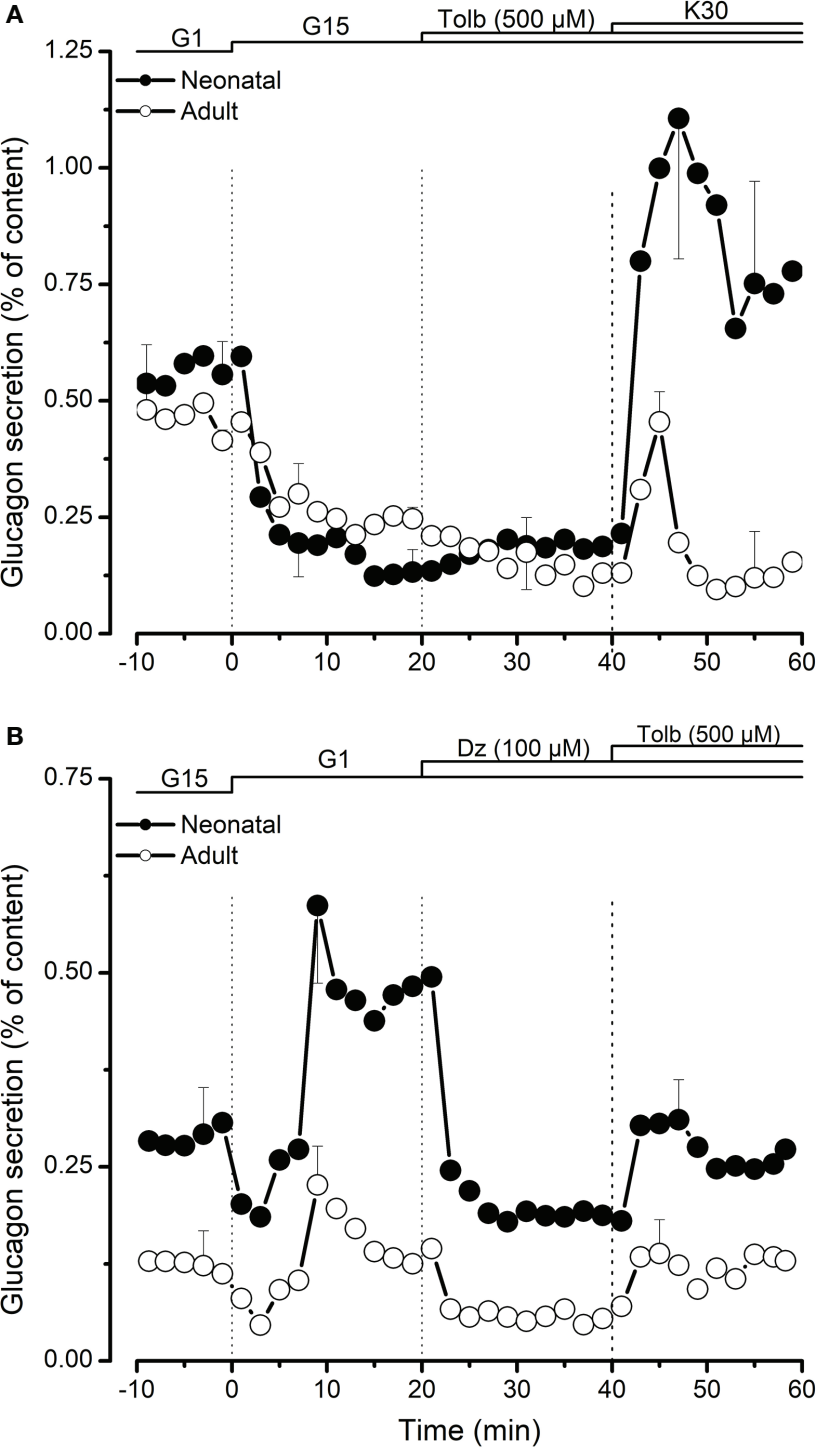
Role of K-ATP channels in regulation of glucagon secretion from neonatal (●) and adult (○) porcine islets. In **(A)**, the effect of K-ATP channel blocker, tolbutamide (Tolb, 500 µM) was studied in the presence of high glucose (15 mM, G15). 30 mM KCl (K30) was added at the end to test the effect of membrane depolarization on glucagon secretion. In **(B)**, the effects of tolbutamide and K-ATP channel activator, diazoxide (Dz, 100 µM) were studied in the presence of low glucose (1 mM, G1). Values are means ± SEM from n=3 different preparations from each age group.

In a second set of experiments, we tested the effect of tolbutamide that mimics the action of glucose on ATP-dependent potassium channels by binding their SUR subunit. In the presence of high glucose (15 mM), 500 µM tolbutamide had no effect on glucagon secretion in both neonatal and adult islets (**Figure 2A**). Membrane depolarization using 30 mM KCl (K30) significantly increased glucagon secretion in the presence of high glucose. As shown in **Figure 2A**, the effect of K30 was significantly higher on neonatal islets compared to its effect on islets from adult pigs. Addition of ATP-dependent channel opener, diazoxide (50 µM) significantly inhibited low glucose-induced glucagon secretion and this inhibitory effect was partially reversed by tolbutamide (**Figure 2B**).

### Effect of cAMP on glucagon secretion

**Figure 3:**
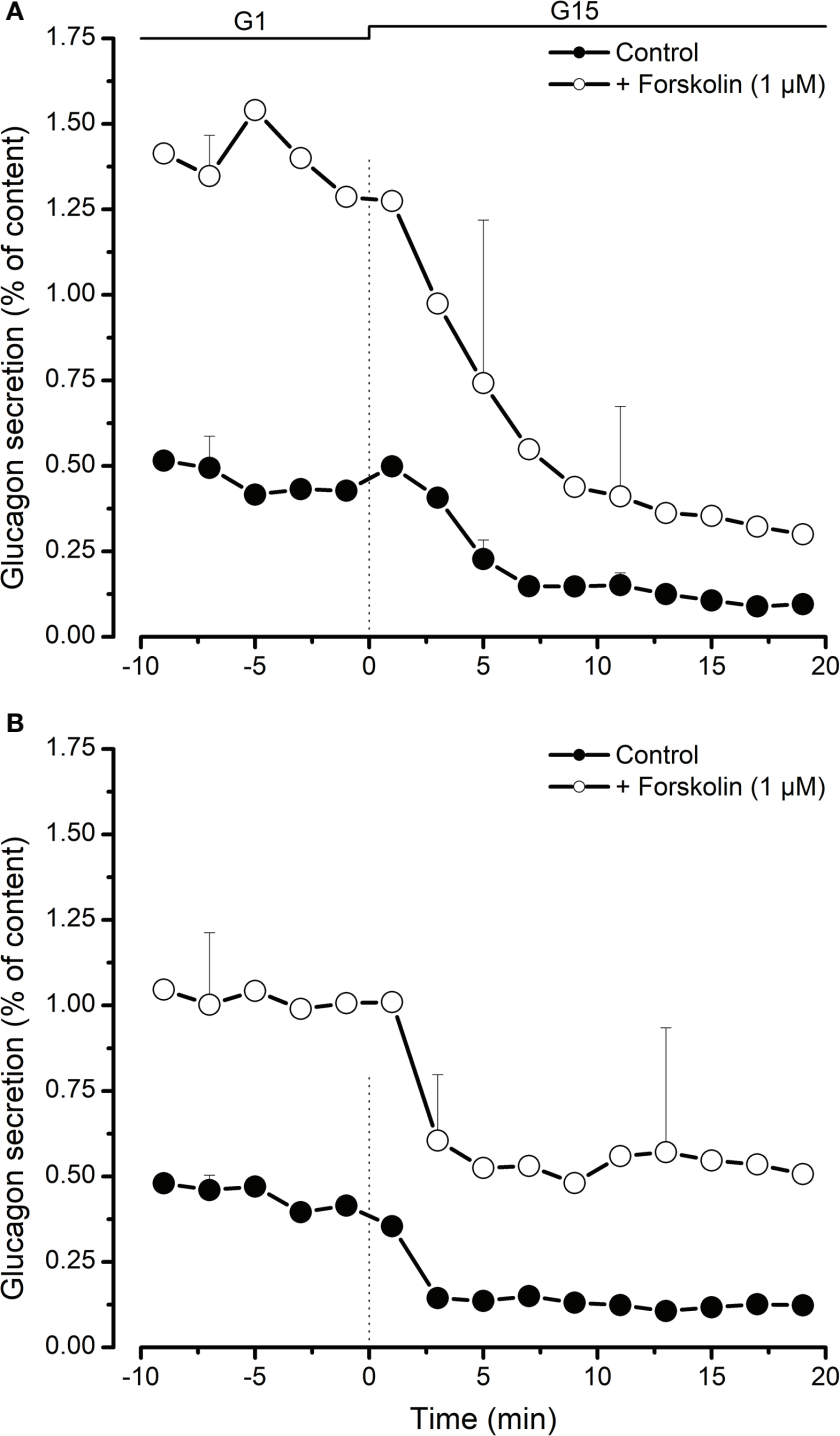
Inhibition of glucagon secretion by high glucose (15 mM, G15) from neonatal **(A)** and adult **(B)** porcine islets in the presence (○) or absence (●) of forskolin (1 µM, adenylyl cyckase activator causing an increase of cytosolic cAMP). Values are means ± SEM from n=3 different preparations from each age group.

We used froskolin (1 µM) to activate c-AMP-dependent secretion pathways in porcine islet cells. As shown in **Figure 3A**, increasing cystosolic cAMP greatly increased stimulated glucagon secretion from neonatal islets and slightly slowed down the inhibitory effect of high glucose. This was also observed, although to a lesser extent, with adult islets (**Figure 3B**).

### *In vivo* response to hyperglycemic stimulation

**Figure 4:**
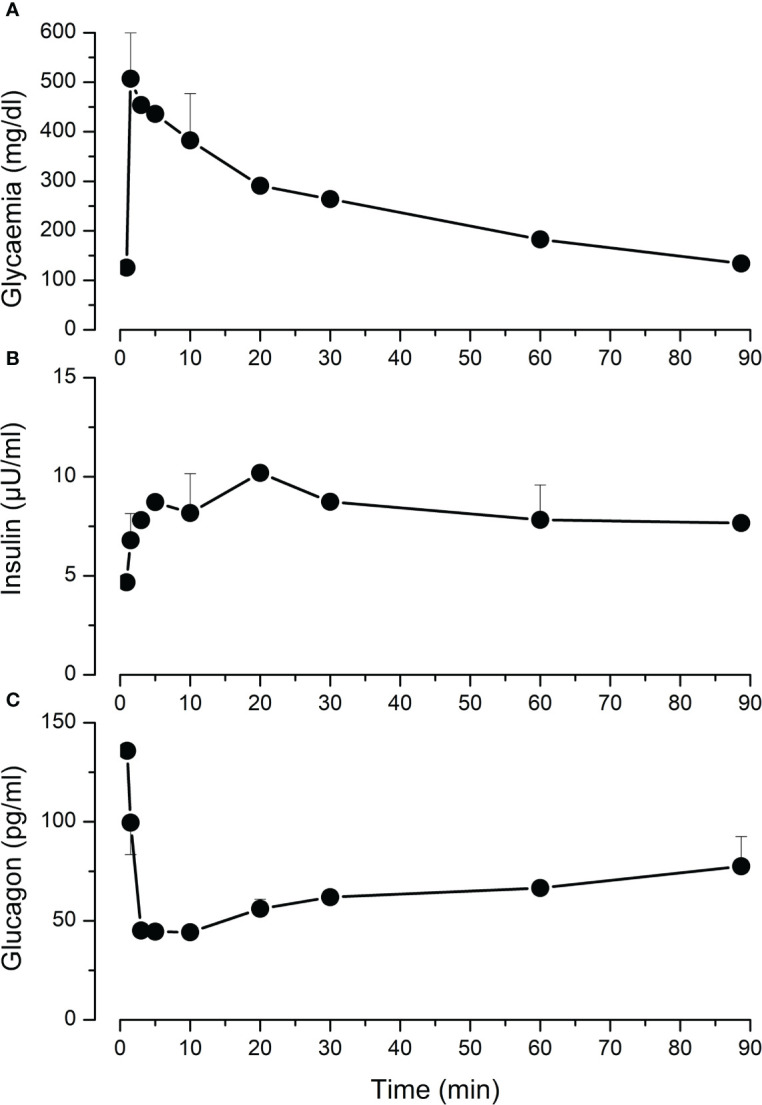
Intravenous glucose tolerance test (IVGGT) in adult pigs. Sedated fasting animals received an intravenous glucose bolus (0.5 g/Kg) at t=0. Blood samples were taken before and during 90 minutes after the injection to measure glycaemia **(A)**, insulin **(B)** and glucagon **(C)**. Values are means ± SEM from n=4 adult pigs.

We performed intravenous glucose tolerance test in 4 adult pigs. Mean fasting glycaemia was 125 mg/dl. Following glucose injection, the rise in glycaemia was detectable already after 2 minutes, and then it gradually decreased and returned to starting values after 90 minutes (**Figure 4A**). Plasma insulin measurement showed a slower response that culminated in a 2-fold increase 20 minutes after glucose injection (**Figure 4B**). In parallel, glucagon concentration dropped 3-fold within 3-5 minutes (**Figure 4C**).

## Discussion

Interest in porcine islets as a potential cell source for islet replacement therapy was sparked by several of their characteristics including physiological compatibility of porcine insulin in humans, availability of theoretically unlimited donor supply and possibility of genetic modifications of donor pigs to render their cells less immunogenic (16, 18, 19), exclude potential xeno-pathogens (20, 21) or even increase their insulin secretion (11). A number of studies have investigated porcine islet physiology both *in vitro* and *in vivo* all agreeing on the low insulin response of neonatal and even adult islets to stimulation with glucose or other secretagogues (11–13, 22). Much less documented is the regulation of glucagon secretion from porcine islets. Our results show that, similarly to rodent (23) and human (24) islets, high concentration of glucose inhibits glucagon secretion from both neonatal and adult islets. In rodent islets, full ATP-dependent potassium channel closure initiated by tolbutamide has been shown to stimulate glucagon secretion in the presence of inhibitory glucose levels (23, 25). In human islets, tolbutamide (100-500 µM), exerts its glucagonostatic effect independently of paracrine activity *via* intrinsic depolarization of alpha-cell membrane and reduction of calcium entry (24, 26). The situation differs in porcine islets since we did not observe any effect of tolbutamide on glucagon secretion when it was already inhibited by glucose. Only membrane depolarization with 30 mM KCl succeeded in stimulating glucagon secretion despite the presence of high glucose. As expected, high concentration of diazoxide (100 µM) inhibited glucagon secretion and this inhibition was partially lifted by tolbutamide in the presence of low glucose. This might reflect membrane depolarization by tolbutamide independently of its effect on action potential amplitude since 100 µM diazoxide completely abolish action potential firing (6). Increasing cytosolic cAMP is known to amplify glucose-stimulated insulin secretion from porcine islets (11, 12). Here, we show that forskolin increases low glucose-stimulated glucagon secretion and slightly reduces the inhibitory effect of high glucose on glucagon. This observation is similar to what has been previously reported in mouse and human islets (27, 28) but differs from other experiments using human islets where GLP-1 and forskolin both induced a 2-3-fold inhibition of glucagon secretion in the presence of 1 mM glucose (29). Compared to their adult counterparts, neonatal porcine islets had greater glucagon content and were more responsive to glucose or tolbutamide stimulation, membrane depolarization and cAMP-dependent amplification even after secretion was reported to total islet glucagon content. Moreover, the effects of these substances on glucagon secretion was more drastic than their effects on insulin secretion especially in neonatal islets suggesting an important role of glucagon in porcine islet physiology. This is supported by our observation that during IVGTT, there was a very rapid 3-fold drop in plasmatic glucagon whereas a 2-fold increase of insulin concentration took 20 minutes to occur. Similarly, a clinical study in healthy individuals showed *in vivo* stimulation of glucagon secretion under hypoglycaemic conditions and inhibition in normoglycaemia or following infusion of tolbutamide. In contrast, the effect of glucose on *in vivo* insulin secretion estimated from plasma C-peptide is more drastic and rapid in humans (30). Differences in pancreatic hormone secretion profiles from neonatal and adult porcine islets reflect maturation of islet cells (31) as previously shown in human islets (32) where basal insulin secretion was high and glucose responsiveness required presence of cAMP-raising agent forskolin. The prominent role of glucagon in neonatal islet physiology might reflect the yet immature state of glucose metabolism-secretion coupling in young beta cells and their reliance on glucagon-dependent cAMP signaling to stimulate insulin secretion in response to glucose (33). In conclusion, our study, the first to investigate regulation of glucagon secretion from porcine islets, highlights the necessity for a better understanding of pig islet physiology as their peculiar secretory characteristics will greatly affect the outcome of their transplantation to human patients.

## Data availability statement

The raw data supporting the conclusions of this article will be made available by the authors, without undue reservation.

## Ethics statement

The animal study was reviewed and approved by Commission d’Ethique pour l’Expérimentation Animale, Secteur des Sciences de la Santé, Université catholique de Louvain.

## Author contributions

NM designed the study, performed experiments, analyzed data and wrote the manuscript. DX performed experiments. PG designed the study and wrote the manuscript. All authors contributed to the article and approved the submitted version.

## Funding

This work was partially funded by the ‘‘Service public de Wallonie, SPW Economie, Emploi, Recherche’’ Grant ‘‘8318 SPW’’.

## Conflict of interest

The authors declare that the research was conducted in the absence of any commercial or financial relationships that could be construed as a potential conflict of interest.

## Publisher’s note

All claims expressed in this article are solely those of the authors and do not necessarily represent those of their affiliated organizations, or those of the publisher, the editors and the reviewers. Any product that may be evaluated in this article, or claim that may be made by its manufacturer, is not guaranteed or endorsed by the publisher.
